# A UK medical student’s view to ‘inclusion of the homeless in health equity curricula: a needs assessment study’

**DOI:** 10.1080/10872981.2020.1832349

**Published:** 2020-10-11

**Authors:** Adam Avnon, Benjamin Anstey, Amy Watts, Emmanuel Obale, Daniel Mclaughlin

**Affiliations:** Faculty of Biology, Medicine and Health, University of Manchester, Manchester, UK

**Keywords:** Homeless, education, health equity, student, curriculum

Dear editor,

As 5th-year medical students in Manchester, we enjoyed reading Feldman et al.’s article exploring attitudes to the inclusion of homelessness in the medical curriculum [1]. Manchester, like many parts of the UK is experiencing a homelessness crisis and our joint involvement in homeless healthcare initiatives meant we wanted to offer our perspective on the matter.

It is well documented that illness can not only precede but also be a consequence of homelessness [2]. The interlink between the two makes the exclusion of homeless healthcare in the medical school curricula particularly salient. This is more noteworthy when accounting for the size of the homeless population; up to 280,000 people are said to be homeless on any given night in the UK [3]. To ignore the healthcare needs of such a large population would seldom be accepted by the medical community.

Perhaps the perceived problematic nature of the homeless population has contributed to the homeless shaped hole in our curriculum.

It was promising to see the positive attitudes of students towards the homeless population, as was the view that it was within the scope of many to aid vulnerable patient advocacy in their future practice. This contradicts current practice in medical education, as many of the vulnerable patient demographics identified in the focus groups, including the homeless population, are currently omitted from the curriculum. Not only is it important to include homelessness on health equity curricula, but efforts must be made to include more vulnerable and peripheral patient groups, if we are to truly realise the NHS mantra of free and fair high-quality healthcare for all.

The preference for case-based learning reflected our own experiences of greater student engagement with case studies during events held by homeless healthcare societies that we have been a part of. Our educational experience was mirrored by the focus group finding that homeless healthcare experience was offered on an elective basis by schools. This is worrying as the voluntary nature of the teaching attests to the attitudes health care professionals have of such patients in practice. To improve this, we would like to advocate for the inclusion of one week of shadowing at a homeless specific general practice (GP), during the mandatory GP block in our medical school teaching. Interactions between students and homeless patients would likely enhance knowledge of the homeless population amongst students, and would provide positive student and patient outcomes. Such positive outcomes were demonstrated in a study which explored supervised student-led smoking cessation clinics aimed at the homeless population [4].

Apprehension about the effects on physical and mental wellbeing of students were an understandable theme found in the focus groups. To ease such fears, medical schools must provide students with appropriate preparation. Our experiences have shown running workshops before exposure to the homeless population have been an effective method of informing students of services they can use if they do experience such effects. Furthermore, these have helped to ease anxieties about challenging consultations and effective communication with this patient-group.

We hope that exposure to homelessness in the medical school curriculum will enable greater inclusion of homeless patients in our healthcare system on three levels. Individual clinician bias towards homeless patients is well documented, thus increased educational exposure may lead to future clinicians who can address their biases and subsequently prove more competent and sympathetic in managing these patients [5]. Secondly, recognising the difficulty homeless patients have navigating our current medical system, may reflect not only the individual deficits of the homeless patients, but also the structural problems of the medical system. Enhanced understanding may encourage future clinicians to implement structural changes which aid easier navigation of the system for homeless patients. Finally, we also hope that more educational experience to homeless patients will translate to increased medical research into this patient demographic.
